# The Evidence Speaks for Itself

**Published:** 1916-07

**Authors:** Julio Endelman


### THE EVIDENCE SPEAKS FOR ITSELF.

BY JULIO ENDELMAN, D.D.S.

In an attempt to survey the status of root canal operations, a writer in a recent issue of the *Dental Summary* * attempts to justify the perpetuation of a system of root-canal therapeutics which, viewed from any angle other than that of the inexperienced observer, must continue, in the light of volumes of confirmative evidence, to be adjudicated as both inefficient and dangerous. The argument is based to some extent on the statement which the writer in question makes to the effect that "when we honestly know that thousands of root canals are filled annually and are not producing infections, but are rendering the services made possible by devitalization," the dental profession should not be made to suffer an unjustifiable opprobrium. Are we to assume from this that the writer contends that the technique of root-canal operations in the past has

* To Save or Not to Save. By James F. Hannon. Reprinted in abstract at page 413 of this issue of The Pacific Dental Gazette.

proven itself to a large extent free from possibilities along the line of disease production in areas of the body remote from the primary seat of injury, and, further, are we to assume that all the clinical, radiographic and bacteriologic evidence of scores of workers is to be relegated to the scrap heap? We believe that no one would be so biased in his conclusion, not even the ultra-radical, as to issue a denial based on careful clinical observations that a proportion of teeth is permanently conserved after removal of the pulp and subsequent filling of the root-canals. That is, of course, a fact beyond argument, and as such it is herein dismissed without further direct comment. It is to the other side of this picture to which our attention shall now be directed; to the army of the physically incapacitated through the constant absorption of bacteria and their toxins from hidden areas of chronic infection in the substance of the jaws, associated with teeth which had been "successfully" treated years previously.

The fact that thousands of teeth remain healthy and useful in appearance and actually do not exhibit any clinical manifestation of chronic infection is *per se* a statement without any argumentative value whatsoever. Any statement whose purport is to approve or disprove clinical conclusions or those which have been reached and framed after a series of careful laboratory experimentation, unless it be based upon statistics covering hundreds, or better yet, thousands of cases, the history of each carefully recorded, is not only valueless, but decidedly dangerous in its possibilities. "In fact," says the essayist in question, "it has been said that there are but a few men in every community who can properly treat and fill root-canals. It would seem, then, that the efforts of the profession, in their study, experience and observations, have been in vain; that, after establishing an aseptic condition in the efforts to maintain this condition, a highly infective condition has been set up by the use of drugs which until this time have rendered very valuable service to dentistry, but alas, it has all been in vain, and we must seek other methods in justice, both to the profession and the patient."

Alas! the efforts have been in vain and have resulted in conditions which the cursory observer is not expected to fully comprehend, and at a cost to the human race which we have at last learned to compute on the basis of permanently lost teeth—nay, on the basis of permanently filled graves. As yet the proportion in communities of those who, being possessed of a pathologic sense, are doing their utmost to prevent the creation in the human mouth of bacterial volcanoes is decidedly small. The number, however, will grow as statistics and laboratory investigations are spread over broader confines and the existence of the empiric in the medical and dental professions is rendered more difficult through the education of the people—the unwitting victims of mistaken notions and falsisms.

Now, in regard to this highly infective condition being set up by the use of drugs which have rendered "very valuable" service to dentistry, we will have to reiterate the statement that we have not been satisfied in the past to kill the bacteria involved with germicides of weaker concentration, but have availed ourselves of such means as not only destroy the bacteria, but also the area of healthy tissue with which the solution or its vapor comes into contact. There is no doubt whatsoever that areas which have been subjected to the action of strong germicides become, in the presence of bacteria, foci of chronic inflammation.

A statistical comparision between the cases of pulp extirpation and subsequent root-canal fillings to whose door could not be traced the cause of systemic infections and those cases which can be grouped as successful operations, constitute a most emphatic argument against the indiscriminate extirpation of pulps and the treatment of root canals on any basis other than that of the strictest of asepsis and in conformity with the findings of those who make it their business to investigate one of the most complicated problems in dentistry in need of exhaustive investigation.

Foci of infection in the mouth are not the only ones responsible for the development of chronic maladies, but when present the problem of correcting these systemic disorders must, among other things, aim at their elimination. The pendulum, it is claimed, has been made to swing, perhaps, too far in the direction of radicalism; in time, however, it is bound to acquire the proper degree of impetus and assume a normal, healthy swing. In the meantime this extreme swaying is helping innumerable victims of empirical methods to discard the yoke of invalidism and to regain their respective places among the useful units in their respective communities.

Any attempt at justifying methods of practice which have been conclusively proven to be inimical to the best interest of the human family, simply because, among other things, it is easier to do poor dentistry or poor medicine than it is to do good dentistry or good medicine, must be condemned in the light of what that poor dentistry leads to, *i. e.*—a series of physical ailments ending, we know, not infrequently, in an untimely journey to lands unknown. Scientific dentistry, the dentistry of whys and wherefores, has become by now sufficiently rooted to insure its permanency; the dentistry of wild guesses and unfounded conclusions is making its exit gradually, but not so fast, however, as to stall off the creeping into our literature of theories deserving of space in volumes of the middle ages, certainly not in the periodical literature of the twentieth century.—*Pacific Dental Gazette.*

